# Health Tract, No. 221

**Published:** 1864-10

**Authors:** 


### HEALTH TRACT, No. 221.

### FOOD FOR CATTLE.

Serious sickness, dyspepsia and a life long train of ills sometimes follow the use of flesh from poor, old, hard worked and diseased animals; it is then of some importance to know how to feed and fatten them properly, and to the best advantage,and to do this the first essential step is to know the relative value, the nutritiousness of various kinds of food, so that the meat when it appears on the table may be fat, healthy, tender and juicy. The following table is the result of carefully conducted experiments made and corroborated by the experiments of eminent chemists and is therefore reliable, as being approximately correct in the main. One hundred pounds of good hay affords as much nourishment to cattle which feed upon it as,

| lbs. | lbs. | lbs. |
|---|---|---|
| 43 of Wheat | 68 Acorns | 195 Boiled Potatoes |
| 44 Dried Peas, | 96 Red clover hay, | 220 Oat straw, |
| 46 " Beans, | 105 Wheat bran, | 262 Ruta baga, |
| 49 " Rye, | 109 Rye bran, | 275 Green corn, |
| 51 " Barley, | 153 Pea straw, | 280 Carrots, |
| 56 " Corn, | 153 Pea chaff, | 339 Man Wurtzel, |
| 59 " Oats, | 167 Wheat or Oat chaff | 346 Field beets, |
| 64 " Buckwheat, | 170 Rye or Barley " | 355 Rye straw, |
| 64 Linseed oil cake, | 175 Raw potatoes, | 504 Turnips. |

### FOOD FOR COWS.

German chemists have found the relative value of food for cows giving milk to be as follows. One hundred pounds of good hay contains as much nourishment as:

| lbs. | lbs. |
|---|---|
| 26 Peas, | 250 Pea straw, |
| 25 Beans, | 300 Barley straw, |
| 50 Oats, | 300 Oat straw, |
| 60 Oil cake, | 350 Siberian cabbage, |
| 80 Clover hay, | 400 Rye straw, |
| 80 Vetches, | 400 Wheat straw, |
| 200 Potatoes, | 460 Beet root with leaves. |

The English give their cows weighing a thousand pounds, eight pounds of good hay, thrice a day in winter. A cow which was given 27 pounds of hay daily, yielded in four days one quart more of milk than when she consumed only 21 pounds of hay; that is, the extra 24 pounds of hay in four days gave one quart of milk extra. While horses require eight per cent. of their weight good English hay a day, milch cows require only two-and-three-quarters per cent. A milch cow will not eat more than 25 or 30 pounds of hay a day, and if more milk is desired, it must be obtained by giving her richer food, that containing more oil, albumen, &c.

